# Systematic review of safety checklists for use by medical care teams in acute hospital settings - limited evidence of effectiveness

**DOI:** 10.1186/1472-6963-11-211

**Published:** 2011-09-02

**Authors:** Henry CH Ko, Tari J Turner, Monica A Finnigan

**Affiliations:** 1 Centre for Clinical Effectiveness, Southern Health, Locked Bag 29, Clayton, Victoria, 3168, Australia; 2 Quality Unit, Southern Health, Locked Bag 29, Clayton, Victoria, 3168, Australia; 3 HK is currently located at the National Health and Medical Research Council Clinical Trials Centre, University of Sydney, Medical Foundation Building, 92-94 Parramatta Road, Camperdown, New South Wales, 2052, Australia; 4 TT is currently located at the Australasian Cochrane Centre, Monash University, Level 6, 99 Commercial Road, Melbourne, Victoria, 3004, Australia

## Abstract

**Background:**

Patient safety is a fundamental component of good quality health care. Checklists have been proposed as a method of improving patient safety. This systematic review, asked "In acute hospital settings, would the use of safety checklists applied by medical care teams, compared to not using checklists, improve patient safety?"

**Methods:**

We searched the Cochrane Library, MEDLINE, CINAHL, and EMBASE for randomised controlled trials published in English before September 2009. Studies were selected and appraised by two reviewers independently in consultation with colleagues, using inclusion, exclusion and appraisal criteria established a priori.

**Results:**

Nine cohort studies with historical controls studies from four hospital care settings were included-intensive care unit, emergency department, surgery, and acute care. The studies used a variety of designs of safety checklists, and implemented them in different ways, however most incorporated an educational component to teach the staff how to use the checklist. The studies assessed outcomes occurring a few weeks to a maximum of 12 months post-implementation, and these outcomes were diverse.

The studies were generally of low to moderate quality and of low levels of evidence, with all but one of the studies containing a high risk of bias.

The results of these studies suggest some improvements in patient safety arising from use of safety checklists, but these were not consistent across all studies or for all outcomes. Some studies showed no difference in outcomes between checklist use and standard care without a checklist. Due to the variations in setting, checklist design, educational training given, and outcomes measured, it was unfeasible to accurately summarise any trends across all studies.

**Conclusions:**

The included studies suggest some benefits of using safety checklists to improve protocol adherence and patient safety, but due to the risk of bias in these studies, their results should be interpreted with caution. More high quality and studies, are needed to enable confident conclusions about the effectiveness of safety checklists in acute hospital settings.

## Background

Patient safety is a fundamental component of good quality health care. Checklists, sometimes referred to as 'safety checklists' or 'medical checklists' are increasingly being suggested as tools to improve care processes and patient safety outcomes. There are suggestions on how to create checklists, what should be in them, and how to implement them in the clinical environment [1]. Checklists might contribute to improved patient safety outcomes, but they are often implemented as a part of multi-component quality improvement initiatives [2]. It has been unclear whether checklists themselves are effective in improving patient safety in acute care settings, and what checklist designs and implementation tools have been used. Southern Health, the largest health service in the state of Victoria, located in Melbourne, Australia, and an integrated health service that includes five hospitals, was considering implementing a patient safety checklist which would be applied to all patients in acute hospital settings by medical care teams. The question posed for this systematic review was whether the use of safety checklists, compared to not using checklists, improves patient safety in acute hospital settings. We are interested in paper-based checklists for this review.

## Methods

### Protocol registration

Because this systematic review was primarily and initially for internal use by Southern Health, and required within a very short timeframe, registration of the protocol was not undertaken.

### Search strategy

We searched the Cochrane Library, MEDLINE, CINAHL, and EMBASE for randomised controlled trials published in English before September 2009 to as early as 1980. The search in MEDLINE was: [(checklist$ OR check-list$ OR ticklist$ OR tick-list$).mp. OR (checksheet$ OR check-sheet$ OR goal-sheet$ OR goalsheet$ OR cognitive aid$ OR cognitive tool$ OR memory aid OR memory tool OR mnemonic).mp.] AND (exp safety/OR exp medical errors/OR exp quality assurance, health care/OR exp risk management/OR (safety OR quality).mp. OR (medic$ AND risk$).mp OR (clinic$ AND risk$).mp). Similar terms were used in the Cochrane Library, EMBASE, and CINAHL. Validated search filters developed by McMaster University's Health Information Research Unit http://hiru.mcmaster.ca/hiru/HIRU_Hedges_home.aspx were used to separate systematic reviews and controlled trials from other types of studies. The full searches are available on request from the authors. Reference lists of included studies were searched for other potentially relevant studies.

### Inclusion and exclusion criteria

The population included all patients in acute hospital settings. The intervention was care given with the use of checklists that addressed safety concerns, which were applied to patients by medical care teams, which had to include a medical clinician or surgeon. The control was care provided without checklists. The outcomes were any patient-relevant clinical outcomes. Studies were excluded if the checklist was not paper-based (i.e. electronic), was part of a multi-faceted quality improvement programme (apart from education provided on how to use the checklist), or was not the primary tool to drive improvements. Only comparative studies written in English since 1980 were considered. Studies were selected and appraised by two reviewers independently in consultation with colleagues.

### Quality assessment and synthesis

The quality of included studies was appraised in detail using the standard critical appraisal questions developed by the Centre for Clinical Effectiveness, Southern Health http://www.southernhealth.org.au/page/Health_Professionals/CCE/ (Table 1). The results from all critical appraisal questions were grouped into seven key study quality areas, and a summary judgement of the overall risk of bias for each area is provided in Table 2. No quantitative meta-analysis was performed.

**Table 1:** Critical appraisal questions for cohort studies used by the Centre for Clinical Effectiveness, Southern Health http://www.southernhealth.org.au/page/Health_Professionals/CCE/

Description of the study
1. Patient/population
2. Number
3. Setting
4. Intervention
5. Comparison/control
6. Outcomes
7. Inclusion criteria
8. Exclusion criteria
Study validity
1. Were there any conflicts of interest in the writing or funding of this study?
2. Does the study have a clearly focused question?
3. Is a cohort study the appropriate method to answer this question?
4. Does the study have specified inclusion/exclusion criteria?
5. If there were specified inclusion/exclusion criteria, were these appropriate?
6. Other than the exposure under investigation, were the groups selected from similar populations?
7. Aside from the exposure, were the groups treated the same?
8. Was exposure measured in a standard, valid and reliable way?
9. Were outcome assessors blind to the exposure?
10. Were all outcomes measured in a standard, valid and reliable way?
11. Were outcomes assessed objectively and independently?
12. Is the paper free of selective outcome reporting?
13. Were the outcomes measured appropriate?
14. Was there sufficient duration of follow up?
15. Was the study sufficiently powered to detect any differences between the groups?
16. If statistical analysis was undertaken, was this appropriate?
17. Were the groups similar at baseline with regards to key prognostic variables?
18. What percentage of the individuals recruited into each arm of the study were lost to follow up?
19. What percentages of the individuals were not included in the analysis?

Other 1. What is the overall risk of bias?

Results

Authors' conclusions

Our comments

**Table 2:** Summary of quality assessment of studies.

Setting	Study	Quality assessment*						
		**No conflict of interest**	**Appropriate study design**	**Appropriate participant selection**	**Appropriate allocation concealment and blinding**	**Appropriate data collection methods used**	**Outcome attributable to intervention**	**Appropriate analysis**

ICU	Agarwal et al 2008 [3]	?	+	+	?	+	+	+

	Berenholtz et al 2004 [4]	++	+	?	-	+	+	+

	Byrnes et al 2009 [5]	++	+	?	?	+	++	?

	Narasimhan et al 2006 [6]	?	+	?	?	+	+	?

	Pronovost et al 2003 [7]	?	+	?	?	+	+	?

ED	Gokula et al 2007 [8]	?	+	+	?	+	+	+

	Romagnuolo et al 2005 [9]	?	+	++	?	+	+	++

Surgery	Haynes et al 2009 [10]	++	+	+	-	+	+	+

Acute care	Weingarten Jr et al 2004 [11]	?	+	+	?	+	?	?

*Quality assessment: ++ = criterion met; + = criterion partially met; - = criterion not met; ? = unclear from the information provided.

### Missing data

Authors of included studies were contacted regarding any questions.

## Results

### Search results

**Figure 1:**
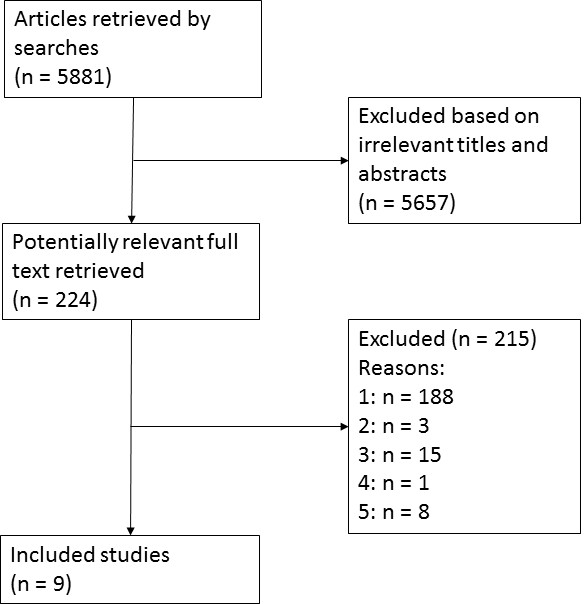
**QUORUM statement flow diagram for medical checklist studies**. Key reasons for exclusion: 1 = not a comparative study; 2 = irrelevant setting; 3 = irrelevant intervention; 4 = irrelevant comparator; 5 = irrelevant outcomes. Search flow chart: n = number of studies.

Due to the volume of articles found using the search string, search filters were used to separate out study types. The systematic review search found 5881 citations, the clinical trial search found 684 citations, and the search for other studies found 3297 citations. Of these, 224 articles were retrieved for full text review and nine cohort studies with historical controls were included. Four clinical settings were covered, including five studies in the intensive care unit (ICU) [3-7]), two studies in the emergency department (ED) [8,9], one study in surgery [10], and one study in multi-departmental acute care [11]. Figure 1 shows the reasons for studies being excluded and included. The studies are summarised in Table 3.

**Table 3:** Summary of included studies and PICO.

Setting	Study	Population	Intervention	Control	Outcomes
ICU	Agarwal et al 2008 [3]	641 paediatric ICU (PICU) patients	Daily patient goal sheet plus education aimed at PICU nurses, paediatric residents, paediatric critical care fellows, and PICU attending physicians	Standard care	Length of stay (LOS) (days)

	Berenholtz et al 2004 [4]	68 surgical ICU (SICU) patients requiring mechanical ventilation	Safety checklist and education aimed at surgeons, and an intensivist-led multidisciplinary team that includes ICU attending physicians and fellows, anesthesia and surgery residents, nurse practitioners, nurses, and a pharmacist	Standard care	Percentage of ventilator days per week when patients received all four care processes of semi-recumbent positioning (for prevention of ventilator-associated pneumonia (VAP)), appropriate sedation, appropriate peptic ulcer disease (PUD) prophylaxis, and appropriate deep venous thrombosis (DVT) prophylaxis

	Byrnes et al 2009 [5]	1285 surgical/burn/trauma ICU patients	Checklist of ICU protocols and objectives requiring verbal review, plus education, aimed at all attending staff and fellows	Standard care	Time from admission until prescription of medical DVT prophylaxis (days), utilisation of physical therapy (%), transferral to telemetry (%), and central catheter duration (days)

	Narasimhan et al 2006 [6]	ICU patients. Number of patients is not reported.	Daily goals worksheet that allows staff to fill in information on various patient care processes	Standard care	LOS

	Pronovost et al 2003 [7]	ICU patients. Number of patients is not reported.	Daily goals form that asks staff to state the tasks to be completed, care plan, and communication plan	Standard care	LOS

ED	Gokula et al 2007 [8]	200 patients of any age admitted to the ED and had an indwelling urinary tract catheter (IUTC) placed in the ED prior to admission to the hospital	Safety checklist used during care, plus education on using the safety checklists aimed at physicians and nurses	Standard care	Presence of an appropriate indication for use of an IUTC, documentation of an indication for IUTC, and a physician order for the IUTC

	Romagnuolo et al 2005 [9]	61 patients whose primary diagnosis was upper gastrointestinal bleeding	Post-endoscopy checklist to be filled out by the endoscopist after ED admission	Standard care	Patient LOS and readmission rates

Surgery	Haynes et al 2009 [10]	7688 patients ≥ 16 years old and who were undergoing non-cardiac surgery. Eight hospitals from eight countries.	19-item surgical safety checklist, which was delivered with educational training, aimed at surgical teams	Standard care	The primary outcome was occurrence of any major complication including death, during the period of postoperative hospitalization, up to 30 days. The secondary outcomes were six safety process measures

Acute care	Weingarten Jr et al 2004 [11]	12 acute-care hospitals across 15 states in the USA. Number of patients unknown.	Medical record checklists, forms and reminders, which were filled out by physicians or nursing staff. Hospitals chose the intervention strategy that suited their institution, so it is likely they were different across hospitals.	Standard care without any checklists, forms or reminders	Proportion of patients receiving antibiotics within eight hours of a diagnosis of pneumonia

### Study quality

The studies were generally of low to moderate methodological quality as summarised in Table 2. Most of the studies either report insufficient information to assess quality or have not met key methodological criteria. Conflict of interest was not reported in most studies, study design was partially explained or open to biases, in some studies methods of participant selection was unclear, allocation concealment and blinding was unclear or quality criteria not met, data collection methods were partially explained, and some studies used partially appropriate analysis methods. These factors increase the likelihood of bias and this means that the results of the studies must be interpreted with caution.

Cohort studies with historical controls are also particularly open to bias, as differences between control and intervention periods in patient population, staffing profile, policies and protocols, etc, may well substantially effect patient outcomes, making the intervention appear more effective than it is.

Completed appraisal tables for the studies can be obtained by contacting the authors.

### Study findings

The results of these studies suggest that overall some improvements in patient safety may result from safety checklists, but the results are not consistent across all studies or for all outcomes. As shown in Table 3, due to the combination of variations in setting, the checklist design and educational training given and outcomes, it was unfeasible to accurately summarise any trends across all studies. Some studies show no difference in outcomes between checklist use and standard care. The key results from individual studies are summarised by clinical setting in Table 4 and below in the text.

**Table 4:** Summary of results for each clinical setting.

Setting	Number of studies	Results	Comments
ICU	5	Different checklists were used, and different outcomes were measured. There was reduction of patient LOS for some studies, and improvements in compliance in some care processes in some studies, but these were not consistent across all studies	The five studies [3-7] all had a high risk of bias

ED	2	Different checklists were used, and different outcomes were measured. Appropriate use of catheters increased following the intervention but was not statistically significant. There was a statistically significant decrease in LOS using the checklist.	The two studies [8,9] had a high risk of bias

Surgery	1	The rate of any complication, surgical-site infection, unplanned reoperation, and death fell significantly with checklist use. The incidence of pneumonia did not improve.	The one study [10] had a moderate risk of bias

Acute care	1	Checklists significantly improved antibiotic administration within eight hours for patients with pneumonia, with patients approximately two times as likely to receive antibiotics within eight hours compared to patients without checklists.	The one study [11] had a high risk of bias

#### ICU setting

The five studies [3-7] that investigated a safety checklist in the ICU setting all had a high risk of bias. Different checklists were used, and different outcomes were measured. The checklists were a paediatric ICU daily patient goal sheet [3], a surgical ICU checklist for mechanical ventilation patients [4], a checklist of ICU protocols and objectives requiring verbal review [5], a daily goals worksheet on various patient care processes such as tests/procedures, medications, and nutrition [6], and a surgical ICU daily goals form that asked staff to state the tasks to be completed, care plan, and communication plan [7]. There was reduction of patient length of stay (LOS) for some studies, and improvements in compliance in some care processes in some studies, but these were not consistent across all studies. Narasimhan et al 2006 [6] reported LOS significantly decreased from 6.4 days in the control period to 4.3 days in the intervention period (p = 0.02). Pronovost et al 2003 [7] reported mean LOS in the control and intervention periods was 2.2 days and 1.1 days respectively. It was not reported if this was statistically significant. In Agarwal et al 2008 [3], there was no statistically significant difference between control and intervention periods on mean or median LOS. Berenholtz et al 2004 [4] reported that the percentage of ventilator days per week when patients received all four care processes (i.e. recumbent positioning (for prevention of ventilator-associated pneumonia (VAP)), appropriate sedation, appropriate peptic ulcer disease (PUD) prophylaxis, and appropriate deep venous thrombosis (DVT) prophylaxis) increased from 30% pre-intervention to 96% during intervention (p < 0.001). The results for the four individual processes all improved between the control and the intervention periods. Prevention of VAP increased from 30% to 96%, appropriate sedation increased from 97% to 100%, appropriate PUD prophylaxis increased from 86% to 100%, and appropriate DVT prophylaxis increased from 92% to 100%. In Byrnes et al 2009 [5], only four domains from the checklist were assessed and compared. There were statistically significantly better results during the intervention period for use of physical therapy (p < 0.0001) and for transfer to telemetry (p < 0.0001). The other two domains of time from admission until prescription of medical DVT prophylaxis (days) and central catheter duration (days) were similar between groups.

#### ED setting

The two studies investigating a safety checklist in the ED setting had a high risk of bias. Gokula et al 2007 used a safety checklist for patients with an indwelling urinary tract catheter (IUTC) [8], while Romagnuolo et al 2005 implemented a post-endoscopy checklist [9]. The outcomes measured were different between studies. In Gokula et al 2007, appropriate use of catheters increased following the intervention but the increase was not statistically significant [8]. Documentation of an indication for a catheter remained unchanged. The presence of a physician order for catheter placement increased significantly from 43% at baseline to 63% post-intervention (OR = 0.44; 95% CI = 0.24, 0.81; p = 0.007). In Romagnuolo et al 2005, there was a statistically significant decrease in LOS using the checklist [9], with median LOS decreasing from 7.0 days in the control period to 3.5 days in the intervention period (p = 0.003).

#### Surgery setting

Hayes et al 2009 used a 19-item surgical safety checklist in eight hospitals in eight countries [10]. The primary outcome was occurrence of any major complication including death, during the period of postoperative hospitalisation, up to 30 days. The secondary outcomes were six safety process measures. It was reported that the rate of any complication at all study locations combined was 11.0% during the control period and fell significantly to 7.0% during the intervention period (p < 0.001). The total in-hospital rate of death dropped from 1.5% to 0.8% (p = 0.003). The overall rate of surgical-site infection declined significantly 6.2% to 3.4% (p < 0.001), as did unplanned re-operation dropping from 2.4% to 1.8% (p = 0.047). There were no statistically significant changes in the rates of pneumonia. The primary outcome of rates of complications also fell when results were analysed to consider income and clustering effects. Rates of complication fell from 10.3% to 7.1% after the intervention among higher-income study locations (p < 0.001) and from 11.7% to 6.8% among lower income study locations (p < 0.001). However, various combinations of outcome measures showed worse patient outcomes across the eight hospital sites [10]. The secondary outcome of process adherence to correct surgical protocols, consisting of six safety measures, showed that the intervention significantly improved five of the six measures (p < 0.001). Only the outcome of ensuring the presence of at least two peripheral intravenous catheters or a central venous catheter before incision in cases involving an estimated blood loss of 500 mL or more did not significantly improve.

#### Acute care setting

In Weingarten Jr et al 2004 [11], hospitals using a checklist administered appropriate antibiotics within eight hours for patients with pneumonia significantly more often than hospitals without the checklist (p = 0.0005). The OR was 1.993 (no CI reported) meaning that patients in hospitals using checklists were approximately twice as likely to receive appropriate antibiotics within eight hours compared patients in hospitals not using checklists.

## Discussion

All nine included studies were cohorts with historical controls. On top of the various methodological biases introduced in each study, other confounders are introduced with these study designs. These include population selection bias due to different time periods, staff selection bias due to different time periods, potential differences in care procedures due to hospitals instigating new safety protocols over time, and unclear reporting and monitoring procedures between periods for outcome assessment, especially in the control period. More high quality studies, such as RCTs, are needed in this area to increase the level of evidence.

Across the studies there were different populations, different time periods for patient enrolment, and different assessments used. There was a lack of details for inclusion/exclusion criteria and baseline population characteristics in most studies. For example, in four of the five studies set in the ICU there was no reporting of specific patient selection criteria [4-7]. Without explicit a priori inclusion and exclusion criteria investigator and staff discretion can play a biased role in population selection. There may be limitations in the generalisability of the studies to non-ICU settings. There was only one study that investigated the use of checklists in diverse socio-economic and surgical settings [10]. In this study the magnitude of the changes in outcomes before and after the intervention varied between study locations. This suggests that the setting may influence the effectiveness of patient safety checklists, and that in locations with good performance at baseline for the measured outcomes there may be limited potential for improvements. The authors noted no effects of income level or surgery type clusters on the outcomes, but geographic location, resource levels, loads on staff, level of staff training, and other factors may have influenced the effectiveness of the intervention [10].

Across studies within each clinical setting, it is hard to summarise and link trends between checklists and outcomes. As shown in Table 3, due to the combination of variations in setting, the checklist design and educational training given, and outcomes, it was unfeasible to accurately summarise any trends across all studies. Even within a particular setting synthesis is challenging. For example in the ICU setting, three studies used checklists to measure LOS outcomes [3,6,7]. However, these studies used different checklist designs, with one form [6] being less descriptive and detailed than the other two checklists [3,7]. The training components of the intervention were not clearly described in many studies. These variations in study design and limitations in reporting prohibit summary of the trends for interventions and outcomes across studies.

It is also important to consider the impact of team and staff factors on the impact of checklists. One study noted that staff changed frequently during the study and was not the same between control and intervention periods [5]. For many of the studies staff changes during the study period could have affected delivery of care, particularly delivering differences in care between treatment groups. However staff turnover is common in hospitals and this may not be something that can be controlled in studies set in hospitals. It is uncertain if other factors (e.g. new policy directives, unit and organisational safety culture) could have influenced the behaviour of staff in caring for patients. One study stated that "there were other efforts during the study period to improve ventilator care and reduce catheter-related infections that may have contributed to reduced LOS" [7].

Most studies did not provide evidence of how they measured if staff were using the checklists properly during their work. All studies used some sort of staff training or education to increase compliance and proper use of the checklists, but it is unclear if the education and training was effective as this was not assessed in any of the studies. Apart from assessors being there to observe staff doing their work and using the checklists, which one study did [5], one did part of the time [10], or allowing other staff to check each other's checklist before proceeding with further actions [4,10,12], there were no other methods to ensure that staff were using the checklists properly. It is also unclear if there is an optimal design of checklists for specific tasks. In most studies the checklist itself was not validated prior to implementation. Validation of the checkilist is important to ensure that the list contains all relevant items, no unnecessary items and that the included items are interpreted accurately and consistently by the users. For example, one study states that "it is not clear that each element of the checklist needed to be there" [5].

There were different outcome measurement periods between treatment groups. The longest period between control and intervention assessments for any study was 12 months [3]. In one study in the ED setting there was a very large difference in the observation periods between the control period (three months) and the intervention period (four weeks) [9].

Outcomes were not uniformly defined across all studies, even relatively well accepted outcomes such as LOS defined and measured in different ways [3,6,7]. Assessment of LOS is also complex as it is not usually normally distributed as assumed in some of the studies, in some studies was measured differently after checklist implementation and the link between LOS and other surrogate outcome measures to the outcome of patient safety is also unclear [4,6]. It is also questionable whether improvements in staff communication and protocol adherence translate directly to improvements in patient outcomes [4,6,9]. It may be incorrect to draw direct links between improved staff communication and protocol adherence and better patient outcomes from any of these studies because we do not know all the characteristics of the patient population that was studied and all other patient care factors. It is also unclear how long after the introduction of the safety checklist outcome assessment should start. One study defined proper checklist use as being when the intervention had been implemented for 60 days [11]. This is an arbitrary point and it is unclear if the 60 day period after the implementation of the intervention can be validly used as a cut-off point. Inclusion of a comprehensive education and training package could increase the optimal use of the intervention earlier. However, studies could also measure outcomes too early and give a false impression of ineffectiveness. The dilemma for healthcare providers is to measure the outcomes as soon as possible, but making sure that the intervention has been properly integrated into clinical practice first. It was unclear if there was a relationship between the effect of using a checklist and time, however most studies only assessed outcomes for a few months. Caution should be exercised when extrapolating any reported short-term outcomes from the studies to longer term predictions about effectiveness. Longer outcome assessments of maybe at least one year, or over a few cycles of staff changes, may be needed to determine the sustainability of changes.

### Strengths and limitations of this systematic review

This review has some limitations. Only comparative studies written in English since 1980 were considered, so potentially relevant studies published in other languages or prior to 1980 may have been missed. We also did not have the resources to hand-search information sources or contact individual hospitals or experts for potentially relevant studies or evaluations of checklist programs. However our search was broad, and we included a wide variety of checklists in a broad range of patient care settings.

The included studies were undertaken in a variety of settings, used varying methods and evaluated differing interventions and outcomes. As a result we were unable to undertake a statistical meta-analysis, however we believe our detailed quality appraisal and narrative synthesis highlights the strengths, weaknesses and key messages of this complex body of literature.

### Implications for further research

Some studies remarked that some of the items in their own checklists were probably unnecessary. To determine the most useful design and content of checklists clinical trials comparing different checklist designs and content within the same settings are required.

There are Cochrane systematic review protocols to review the evidence on the effect of computer-generated paper reminders [13] and paper reminders on practice and healthcare outcomes [14] but these are looking at a broader range of interventions and outcomes. A more recent published evaluation of the WHO surgical checklist was published in 2010 [15]. There has been a retrospective publication of an 8 year use of safety checklists in neurosurgery [16], and we hope this will result in more publications of long-term comparative studies in this area, both retrospective and prospective studies. In conjunction with this systematic review, Southern Health designed and implemented a medical safety checklist for use by clinical staff and has been monitoring its effect on clinical outcomes. Results from this pilot work at Southern Health may help inform the body of evidence for using safety checklists to improve safety. Concurrently, Southern Health is looking at piloting the use of electronic checklists in improving patient safety. Southern Health plans to update this current systematic review in 2012, and add an appraisal of the evidence for electronic checklists.

### Implications for practice

Health services planning to implement safety checklists should use an evidence-based approach to selecting or designing and validating checklist and/or checklist items for their clinical improvement goals. Resource use should also be considered, such as staff time and funding requirements to properly provide training and education for using the checklists. Health services piloting new checklists or using established checklists should be encouraged to create an evaluation plan on their use of safety checklists and publish their findings so that the body of evidence can grow.

## Conclusions

From nine studies in four hospital care settings, there was no high level evidence showing the effectiveness of safety checklists. These studies suggest some benefits of using safety checklists to improve protocol adherence and patient safety, but the studies had a moderate to high risk of bias so their results should be interpreted with caution. More high quality and high level studies, are needed to enable more confident conclusions about the effectiveness of safety checklists in acute hospital settings.

## Competing interests

The authors declare that they have no competing interests.

## Funding

This systematic review was done as part of normal operational services provided to Southern Health by the Centre for Clinical Effectiveness. No additional funding was received.

## Authors' contributions

MF requested the systematic review from the Centre for Clinical Effectiveness and provided clinical expertise and interpretation. With assistance from MF, HK and TT developed the search strategy. HK applied inclusion criteria to search results in consultation with TT. HK appraised the nine included papers. TT was an independent second reviewer for all included papers. HK prepared the first draft of this article which TT and MF then reviewed. All authors read and approved the final manuscript.

## Pre-publication history

The pre-publication history for this paper can be accessed here:

http://www.biomedcentral.com/1472-6963/11/211/prepub
